# THE ROLE OF CANCER-RELATED FATIGUE IN PERCEIVED AND ACTUAL ATTENTION AMONG OLDER COLORECTAL CANCER SURVIVORS

**DOI:** 10.1093/geroni/igae098.4092

**Published:** 2024-12-31

**Authors:** Mi Sook Jung, Kyeongin Cha, Ahrim Lee, Xirong Cui, Dahyeon Lee, Jeongeun Hwang, Eunyoung Park, Hyun-E Yeom

**Affiliations:** Chungnam National University, Daejeon, Republic of Korea; Chungnam National University, Daejeon, Republic of Korea; Chungnam National University, Daejeon, Republic of Korea; Chungnam National University, Daejeon, Republic of Korea; Chungnam National University, Daejeon, Republic of Korea; Chungnam National University, Daejeon, Republic of Korea; Chungnam National University, Daejeon, Republic of Korea; Chungnam National University, Daejeon, Republic of Korea

## Abstract

Older people treated for cancer often experience greater fatigue compared to younger survivors, partly due to age-related physiological changes and reduced physical resilience. Our previous study found that both perceived cognition and actual test performance were significantly associated with cancer-related fatigue (CRF) in middle-aged cancer survivors. However, it remains unclear whether the impact of fatigue on cognition is more pronounced in older cancer survivors compared to their younger counterparts. This study aims to compare attention function between different age groups and to examine whether increased fatigue contributes to poor attention. We assessed middle-aged (n=131) and older (n=133) colorectal cancer survivors using self-rated questionnaires (Attentional Function Index, Functional Assessment of Cancer Therapy-Fatigue, Patient Health Questionnaire) and a computerized test (Attention Network Test, ANT). Data were analyzed using multivariable linear regression and linear mixed models. Older participants exhibited poorer executive attention performance than middle-aged participants, while perceived attention and fatigue levels were similar between age groups. Additionally, older participants did not show improvement across trials unlike middle-aged participants showing faster performance with repeated trials. Fatigue was significantly associated with performance on ANT. This association remained significant even when controlling for educational level, comorbid condition, depressed mood, and restorative activity. The findings indicate that older cancer survivors had reduced executive attention function and may be more vulnerable to the effect of cancer-related fatigue, which is intrinsically linked to attention dysfunction. This enduring challenge suggests a need for targeted intervention addressing both cancer-related fatigue and cognitive impairment to improve outcomes for older cancer survivors.

